# Supplementary material for: Splice-Junction-Based Mapping of Alternative Isoforms in the Human Proteome
Source: Cell Rep. Author manuscript; Available in PMC 2020 Jan 15. (PMC6961840; doi:10.1016/j.celrep.2019.11.026)
Supplement: 2 [file NIHMS1546469-supplement-2.zip › DF1/PXD006675/RightAtrium/RightAtrium_26_TPM3_AISEELDHALNDMTSG.pdf]

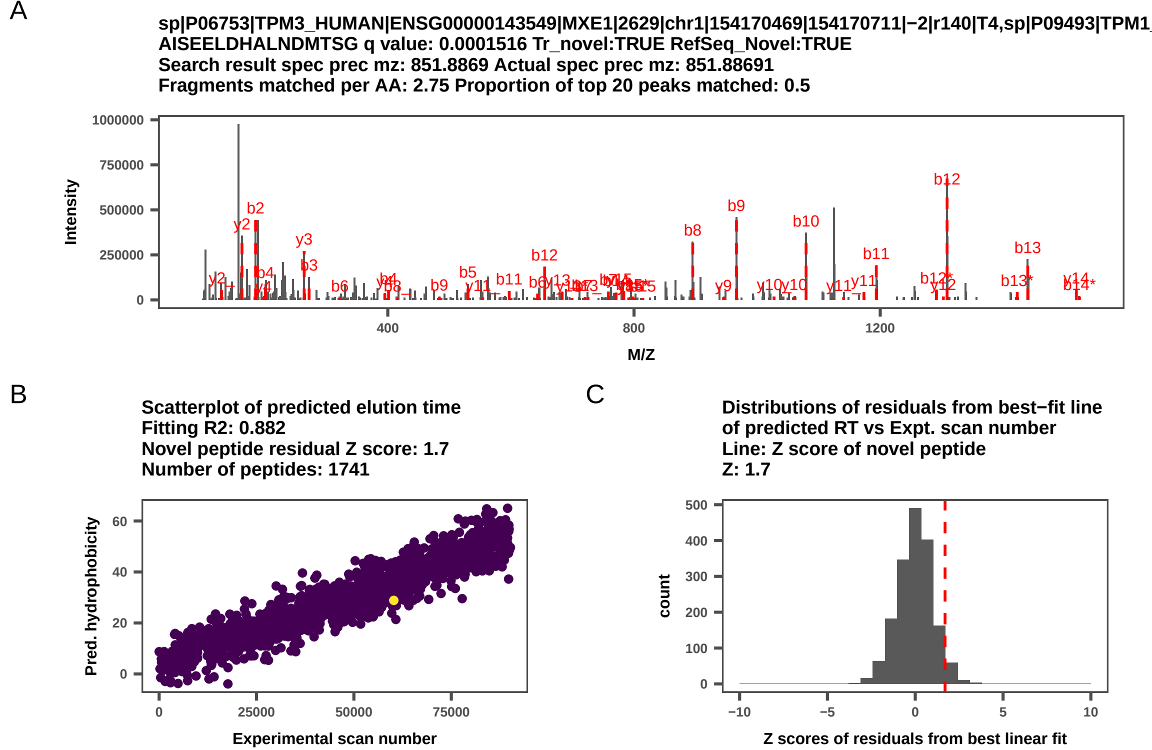
A
sp|P06753|TPM3_HUMAN|ENSG00000143549|MXE1|2629|chr1|154170469|154170711|−2|r140|T4,sp|P09493|TPM1_
AISEELDHALNDMTSG q value: 0.0001516 Tr_novel:TRUE RefSeq_Novel:TRUE
Search result spec prec mz: 851.8869 Actual spec prec mz: 851.88691
Fragments matched per AA: 2.75 Proportion of top 20 peaks matched: 0.5
Intensity
1000000
750000
500000
250000
0
M/Z
400
800
1200
B
Scatterplot of predicted elution time
Fitting R2: 0.882
Novel peptide residual Z score: 1.7
Number of peptides: 1741
Pred. hydrophobicity
Experimental scan number
0
25000
50000
75000
C
Distributions of residuals from best−fit line
of predicted RT vs Expt. scan number
Line: Z score of novel peptide
Z: 1.7
count
Z scores of residuals from best linear fit
